# Pickering Emulsions Electrostatically Stabilized by Cellulose Nanocrystals

**DOI:** 10.3389/fchem.2018.00409

**Published:** 2018-09-19

**Authors:** Swambabu Varanasi, Leeav Henzel, Llyza Mendoza, Ragesh Prathapan, Warren Batchelor, Rico Tabor, Gil Garnier

**Affiliations:** ^1^Department of Chemical Engineering, Bioresource Processing Research Institute of Australia, Monash University, Clayton, VIC, Australia; ^2^Department of Chemical Engineering, Indian Institute of Petroleum and Energy, Visakhapatnam, India; ^3^School of Chemistry, Monash University, Melbourne, VIC, Australia

**Keywords:** cellulose nanocrystals (CNC), Pickering emulsions, oil in water, gels, electrostatic stabilization

## Abstract

Cellulose Nanocrystals (CNC) are explored to stabilize oil/water emulsions for their ability to adsorb at the oil/water interface. In this work, the role of electrostatic forces in the CNC ability to stabilize oil/water emulsions is explored using canola oil/water and hexadecane/water as model systems. Canola oil/water and Hexadecane/ water (20/80, v/v) emulsions were stabilized with the addition of CNCs using ultrasonication. Emulsion droplet sizes range from 1 to 4 μm as measured by optical microscopy. It is found that CNC can stabilize oil/water emulsions regardless of their charge density. However, reducing the surface charge density, by adding salts and varying pH, can reduce the amount of CNC's required to form a stable emulsion. Just by adding 3 mM Na^+^ or 1 mM or less Ca^+2^ to a CNC suspension, the amount of CNC reduced by 30% to stabilized 2 mL of Canola oil. On the other hand, adding salt increases the emulsion volume. The addition of 100 mM Na^+^ or the reduction of pH below 2 leads to the aggregation of CNC; emulsions formed under these conditions showed gel-like behavior. This work shows the potential of nanocellulose crystal in stabilizing food and industrial emulsions. This is of interest for applications where biodegradability, biocompatibility, and food grade requirements are needed.

## Introduction

Cellulose is the most abundant natural polymer. It consists of anhydroglucose units linearly linked through β-1,4-glycosidic bonds. Cellulose nanocrystals (CNC) and cellulose nanofibers (CNF) are two types of exciting green nanomaterials prepared from cellulosic sources (Safari et al., 2014). Nanocellulose has recently been investigated for a multitude of applications because of their renewable nature and unique properties such as wettability, large surface area, high aspect ratio, biocompatibility, being optically transparent, and amphiphilic nature, their strength and ease of chemical modification (Hosseinidoust et al., 2015; Trache et al., 2017). CNC particles are processed from native cellulose sources by controlled acid hydrolysis (Moon et al., 2011). Sulfuric acid hydrolysis dissolves the amorphous fraction, releasing the cellulose nanocrystals (CNC) with sulfate ester groups surface, resulting in electrostatically stabilized aqueous suspensions of CNC (Dong and Gray, 1997). In a dilute aqueous system, CNC particles are well dispersed and orient randomly due to electrostatic repulsion among negatively charged sulfate ester groups (Dong and Gray, 1997). The surface charge of CNC is primarily controlled by the hydrolysis conditions. Higher acid concentration, reaction time and temperature produces CNC of higher surface charge as promoted by sulphuric acid diffusion into fibers (Nishio et al., 2016). Zeta-potential of CNC produced by sulphuric acid hydrolysis varies from −20 to −80 mV. In contrast, if hydrochloric acid is used to produce CNC, the resulting CNC has almost no charge (Nishio et al., 2016).

Many types of nano or microscale particles are used to stabilize the oil/water interface of emulsions, commonly known as Pickering emulsions (Wu and Ma, 2016). Pickering emulsions possess many unique features over classical surfactant stabilized emulsions, such as low toxicity and superior stability (Yang et al., 2017). Particle size, shape, wettability, surface properties, and particle concentration all influence the stability and drop size of emulsions (Wu and Ma, 2016). A key factor to form Pickering emulsions is wettability, which can be indicated by the contact angle at the interface. Binks et al. reported that particles of very low hydrophilicity or very high hydrophobicity are not suitable to form stable emulsions (Binks and Lumsdon, 2000). Particles having an intermediate contact angle (close to 90°) can easily accumulate at the oil/water interface and form stable emulsions (Binks and Lumsdon, 2000). The optimum contact angle to prepare stable oil/water emulsion is around 70° and water/oil emulsion is 110° (Kaptay, 2006). Particles having smaller size will have faster adsorption kinetics and more efficient packing at the oil/water interface than bigger particles (Wu and Ma, 2016). Particle size should be at least one order of magnitude smaller than droplet size to prepare stable emulsion (Wang, 2013). Cylindrical or elliptical shaped particles showed superior stability than spherical shape particles since cylindrical particles pack like network structure and also their ability to shape capillary force between absorbed particles at the interface is superior (Dugyala et al., 2013; Wang, 2013). In order to keep large surface area particles stable in suspension, there must be a steric hindrance or electrostatic repulsion between the particles. However, this force between particles acts as an activation barrier for particle adsorption. At present, there is very little information in the literature to describe the governing laws between surface charge and particle adsorption of CNC (Wu and Ma, 2016).

In this study, CNCs were investigated to prepare very strong and stable Pickering emulsions because of their high aspect ratio. Cellulose Nanofibers (CNF) are also being used in Pickering emulsions (Kalashnikova et al., 2011; Capron and Cathala, 2013; Carrillo et al., 2015; Wang et al., 2016; Capron et al., 2017). Although both CNCs and CNFs are not surface active, they position efficiently at the oil/water or water/oil interface because of their amphiphilic nature (Capron et al., 2017). A presence of hydrophobic edge plane is attributed to its amphiphilic nature. Kalashnikova et al. first reported preparing Pickering emulsions with bacterial CNC (Kalashnikova et al., 2011). Later they described that when sulphated cotton cellulose nanocrystals (CNC) were used, no emulsion was observed (Kalashnikova et al., 2012). To prepare Pickering emulsions with sulphated CNC, the surface charge density has to be modulated to 0.033 e/nm^2^ or lower. The charge density of CNC can be modulated by treating sulphated CNC with mild HCl or adding salts. However, thermodynamically amphiphilic particles can form stable emulsions regardless of their charge density. Many reports concluded that the surface charge of CNCs and CNFs play a key role in the stability of emulsions (Marinova et al., 1996; Kalashnikova et al., 2011, 2012; Fujisawa et al., 2017; Miao et al., 2017). However, there is no systematic study on the role of electrostatics in CNC based emulsions.

CNCs are also being used for preparing high internal phase emulsions (HIPE) (Marinova et al., 1996; Fujisawa et al., 2017; Miao et al., 2017). Capron and Cathala reported a two-step procedure to prepare HIPEs with CNCs (Fujisawa et al., 2017). First, a low internal phase emulsion having 10% oil content was prepared using an ultrasonicator. In the second step, extra oil was added into the already formed emulsion, followed by shearing with a double cylinder-type homogenizer. The resulting final emulsions had a gel-like appearance. Miao et al. also reported another type of two-step procedure to prepare HIPEs with CNC (Marinova et al., 1996). In their study, homogenization at low shear (approximately 2,000 rpm for 1 min) was followed by high shear (10,000 rpm for 1 min). However, the viscoelastic properties of these gel-like emulsions were not reported.

This study aims at exploring whether stable emulsions can be prepared from CNCs having surface charge density higher than 0.033 e/nm^2^ and if modulating their charge density, by adding salts and varying pH, can reduce the amount of CNC's required to form a stable emulsion. This paper investigates the role played by electrostatic forces in CNC based emulsions and whether these are completely charge driven systems. This study also describes a method to prepare CNC based on emulsified gels on a single shearing method.

## Experimental section

### Materials

Cellulose nanocrystals (CNC, 12.6 wt %) were purchased from the University of Maine Process Development Center as a dispersion in water (sulfur content of 1–2%), with an average width of 8 nm and length 138 nm. Canola oil was purchased from the local supermarket. Hexadecane, Sodium Chloride (NaCl) and Calcium Chloride (CaCl_2_) were purchased from Sigma Aldrich, Australia. Oils were used as supplied- without further purification.

#### Methods

1. **Emulsion preparation:** All the emulsions were prepared using an oil/water ratio of 20/80 (v/v). Typically, 2 mL of canola oil was added to 8 mL of the CNC aqueous suspension in a 50 mL vial and was sonicated with a titanium probe (Sonics Vibra-cell High Intensity Ultrasonic Processor, VCX 500–VCX 750) immersed in the solution under the following conditions: 3 s on and 3 s off for an interval of 3 min, with 44 Watts power input. The sonicated emulsions were then poured into 15 mL centrifuge tubes (without any separation) and centrifuged at 4,000G for 10 min to estimate the stability of CNC-palm oil emulsion. Cream volume after centrifugation is noted as emulsion volume.
2. **Conductometric Titration:** Conductometric titration was performed to determine the surface charge density as reported in Kalashnikova et. al. (Kalashnikova et al., 2012). In brief, a total of 50 mL of a CNC suspension at 1 g/L in water was titrated with freshly prepared 2 mM NaOH with a TIM900 titration manager and a CDM230 conductimeter equipped with a CDC749 titration cell (Kalashnikova et al., 2012; Zhong et al., 2012). CNC samples showed low conductance values because of their small amounts of charge so HCl and NaCl were added prior to titration.
3. **Z-potential measurement:** The electrophoretic mobility of aliquots of aqueous CNC suspensions at 0.1 g/L was measured in triplicate with a NanoBrook Omni (Brookhaven's Instruments).
4. The optical micrographs of the prepared emulsions on transparent glass slides were taken using a microscope (Nikon upright motorized microscope Eclipse Ni-E) immediately after each preparation. Drop sizes and distribution were measured using ImageJ software.
5. **Rheology:** All rheological testing of the gel like emulsions were performed with an Anton Paar MCR302 rheometer. A cone (0.997°) and plate (49.975 mm) geometry were selected. Testing was done at ambient temperature (25°C). To ensure stable temperature during the testing, a solvent trap was used. Viscosity was measured at shear rate ranging from 0.5 to 100 s^−1^. Oscillatory strain sweep was performed from 0.01 to 100% at a constant 1 Hz frequency. Frequency sweep was measured from 0.1 to 100 rad/s and at 0.1% strain. All measurements were in triplicates.
6. **Atomic Force Microscopy imaging:** Atomic force microscopy (AFM, JPK Nanowizard 3) was used in alternating contact, AC mode to obtain images of CNC morphology and particle size. CNC suspension drop was casted and air died on glass slide.

## Results

**Figure 1:**
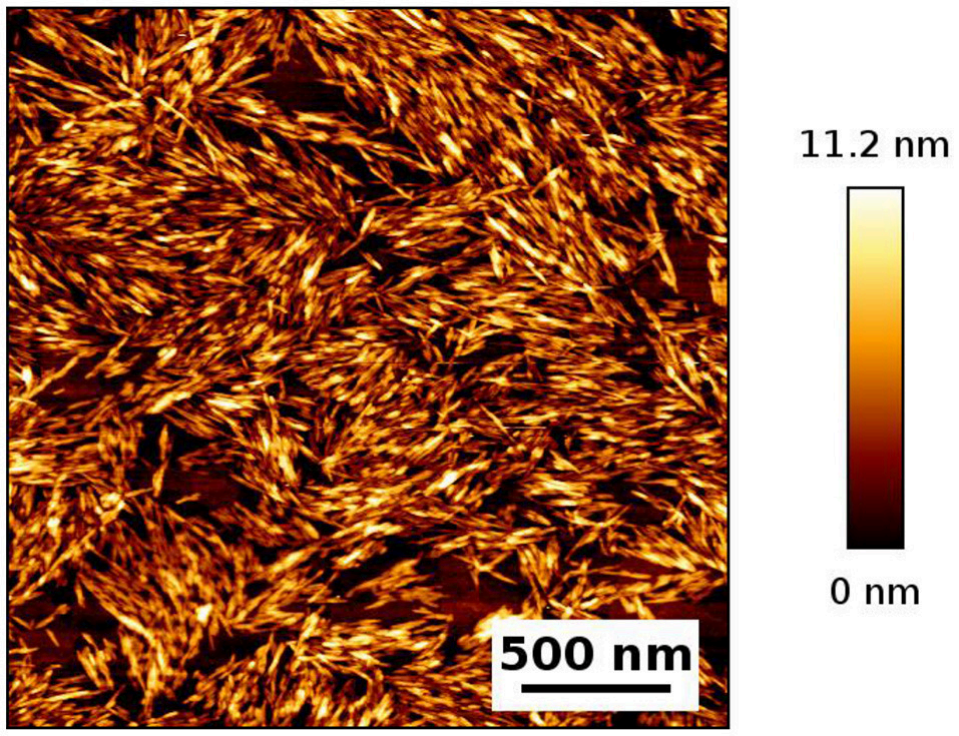
AFM Image of CNC deposited on a glass surface and dried.

**Figure 2:**
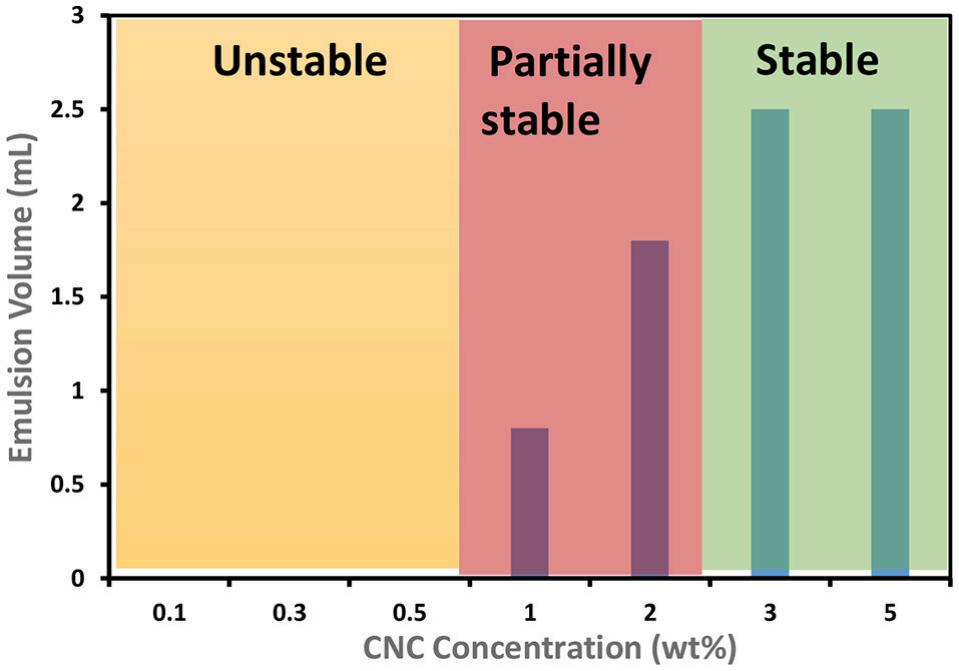
Effect of CNC concentration on stability of emulsions prepared with canola oil and hexadecane (oil to water ratio is 2:8 mL) at room temperature, pH = 7.

An AFM image of the CNC studied is shown in Figure 1. Cellulose nanocrystals (CNC) having an average diameter 8 nm, a length 138 nm, a surface charge density of 0.11 e/nm^2^ and a zeta potential of −70 mV were used to prepare a series of canola oil (C. Oil)/water and hexadecane/water emulsions. The CNC rods exhibit a strongly negatively charged surface because of the presence of hydroxyl and sulfate groups. CNC concentration was varied to study this effect on the stability of oil/water emulsions prepared with two types of oils. Results are shown in Figure 2. Hexadecane behaves similarly to C. Oil. CNCs could not stabilize the emulsions at concentrations ranging from 0.1 to 0.5 wt%. This supports the work of Kalashnikova et.al also reporting that CNC could not stabilize hexadecane/water emulsion at a concentration of 0.1 wt% (Kalashnikova et al., 2012). Interestingly, CNC stabilizes emulsions at a concentration higher than 1 wt% CNC for both types of oils, even though the CNC surface charge density is higher than 0.033 e/nm^2^. Canola oil/water and hexadecane/water samples were centrifuged at 4,000G for 10 min to test their stability.

**Figure 3:**
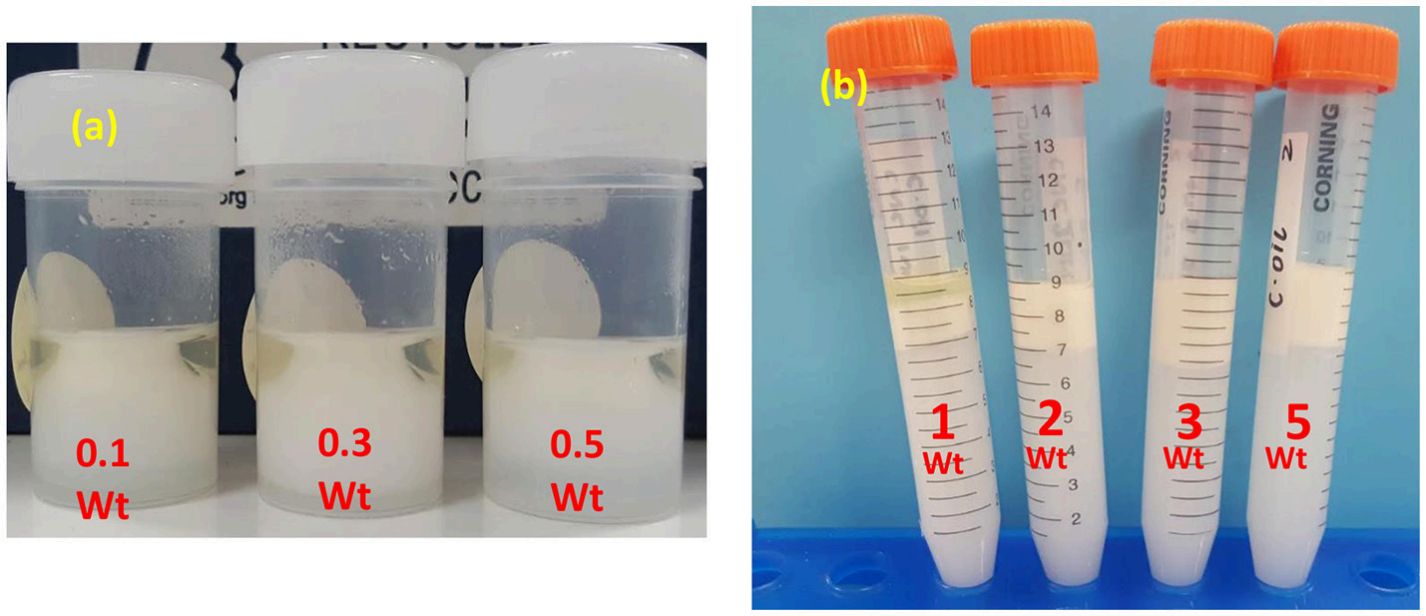
Photographs of CNC stabilized C. Oil/water emulsion after centrifugation. **(a)** Low CNC concentrations and **(b)** high CNC concentrations.

Figure 3 shows the appearance of C. Oil/water emulsions after centrifugation in vials and centrifuge tubes. Emulsions were prepared with CNC concentration ranging from 0.1 to 0.5 wt% (Figure 3a) and from 1 to 5 wt% (Figure 3b). The oil and aqueous layers separated clearly. At CNC concentrations of 1 and 2 wt%, a stable emulsion is formed between the oil and the aqueous layers where the emulsion volume is lower than the initial oil volume. In contrast, at CNC concentrations of 3 and 5 wt%, stable emulsions and aqueous layers were observed, with the emulsion volume higher than the initial oil volume. Completely stable emulsions are formed at 3 wt% CNC and higher concentrations.

**Figure 4:**
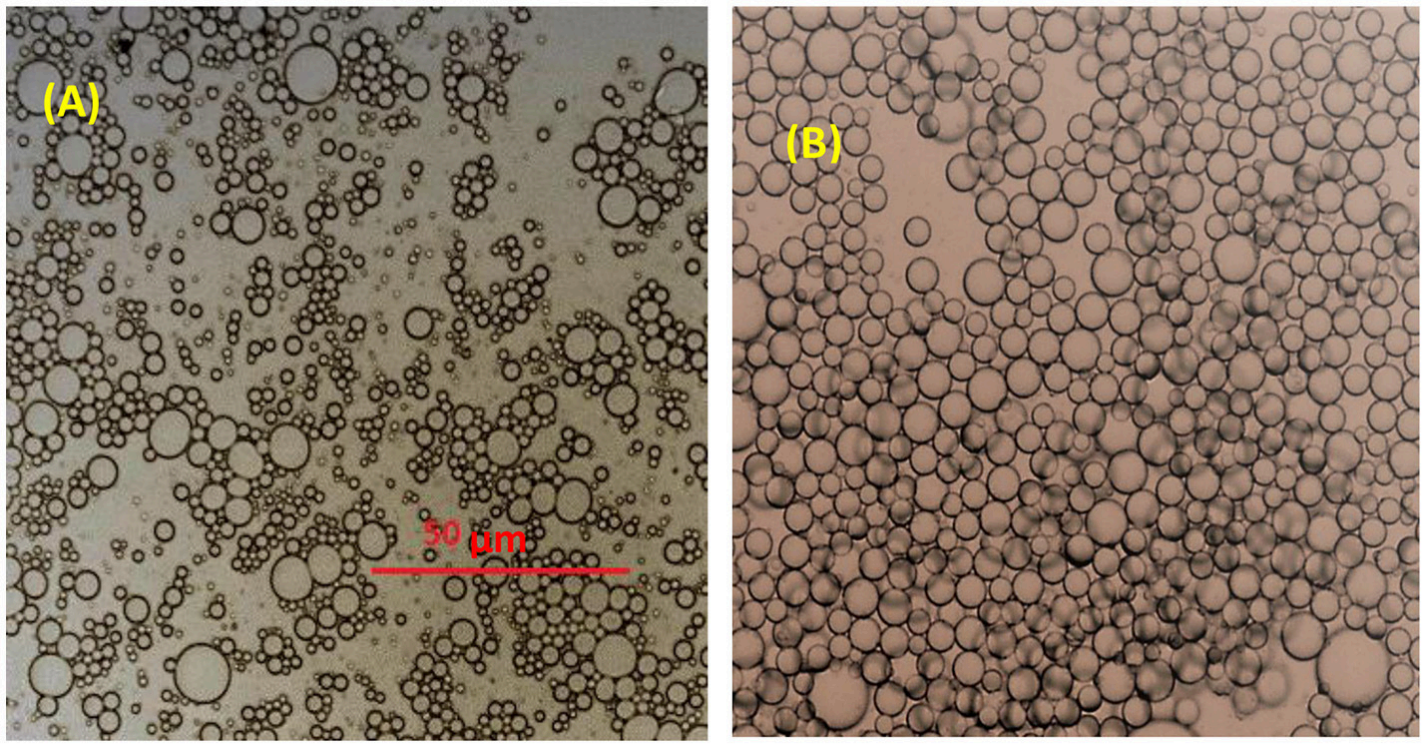
Microscopic image of emulsions stabilized with 3 wt% CNC **(A)** C. Oil/water **(B)** hexadecane/water emulsions.

**Figure 5:**
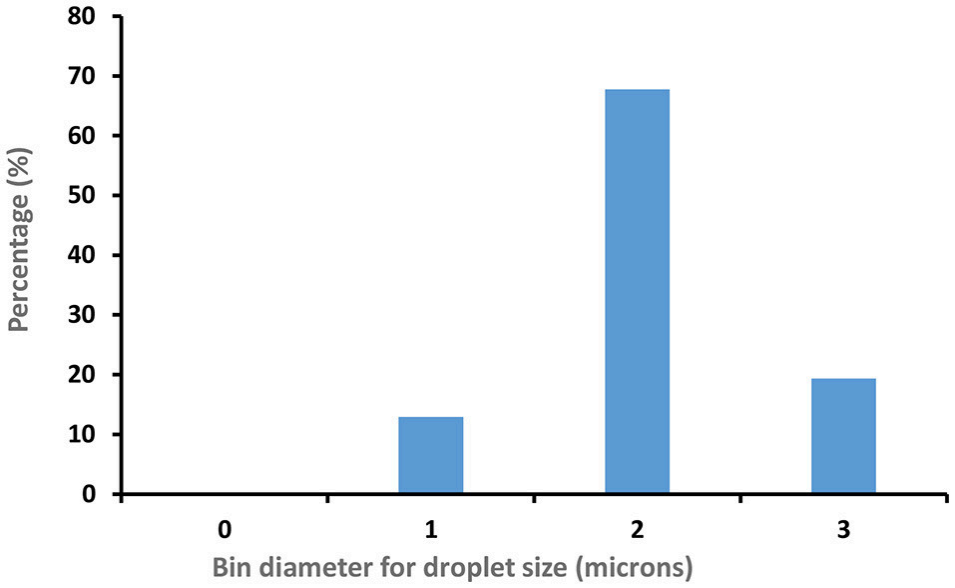
CNC (3 wt %) stabilized Hexadecane/water Emulsion droplet size distribution (100 droplets measured).

**Figure 6:**
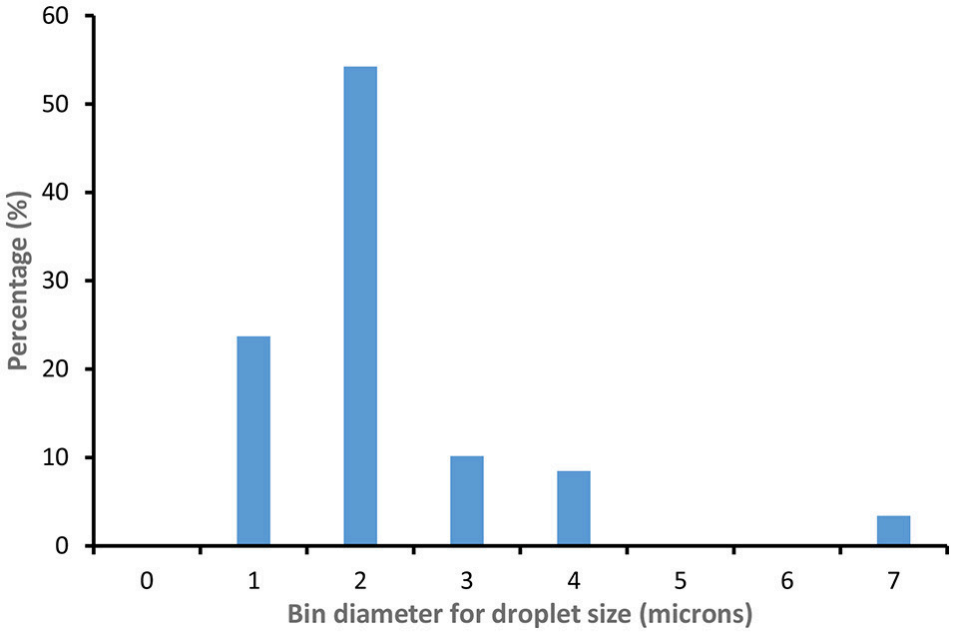
CNC (3 wt %) stabilized C. Oil/water Emulsion droplet size distrubution (100 droplets measured).

Figure 4 displays optical microscopy images of CNC stabilized C. Oil and hexadecane in water emulsions. Droplets of hexadecane/water emulsion are uniformly dispersed, with an average diameter of 2.5 μm (std. dev−0.3 μm) microns (Figure 5). However, C. Oil/water emulsions with an average diameter of 2.7 μm and a high standard deviation of 1.2 μm (Figure 6) are more polydispersed and have random aggregates.

### Effect of salts on emulsions stability

**Figure 7:**
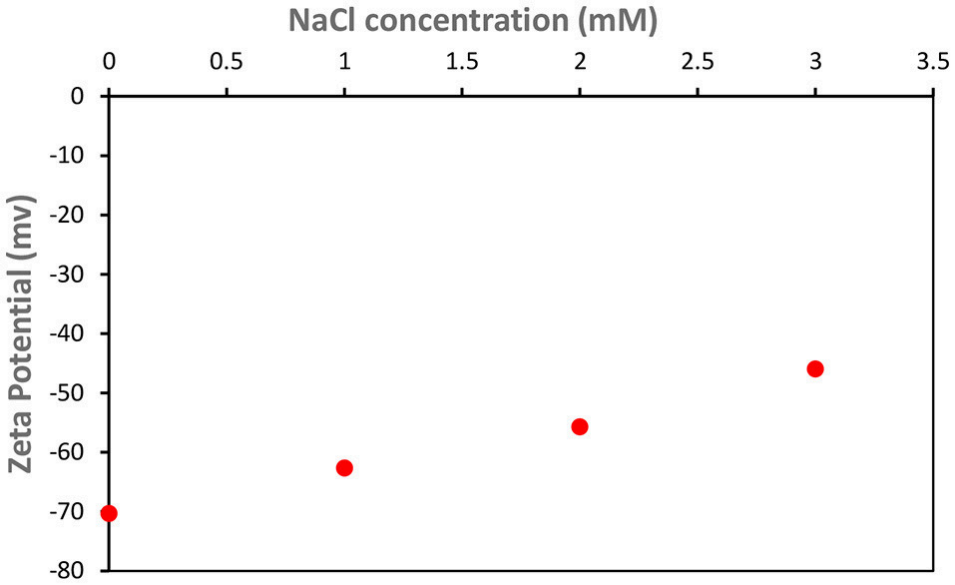
Effect of NaCl addition on the zeta potential of CNC Suspension at pH = 7 and room temperature.

**Figure 8:**
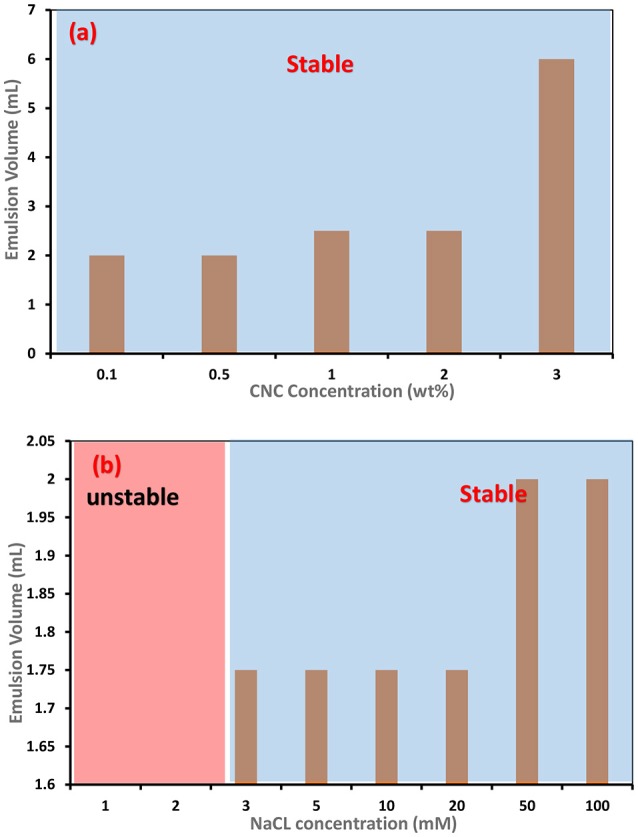
Effect of NaCl addition on the stability of C. Oil/Water emulsions– **(a)** CNC concentration varied keeping NaCl concentration constant at 100 mM **(b)** NaCl concentration varied keeping CNC concentration constant at 0.1 wt.

**Figure 9:**
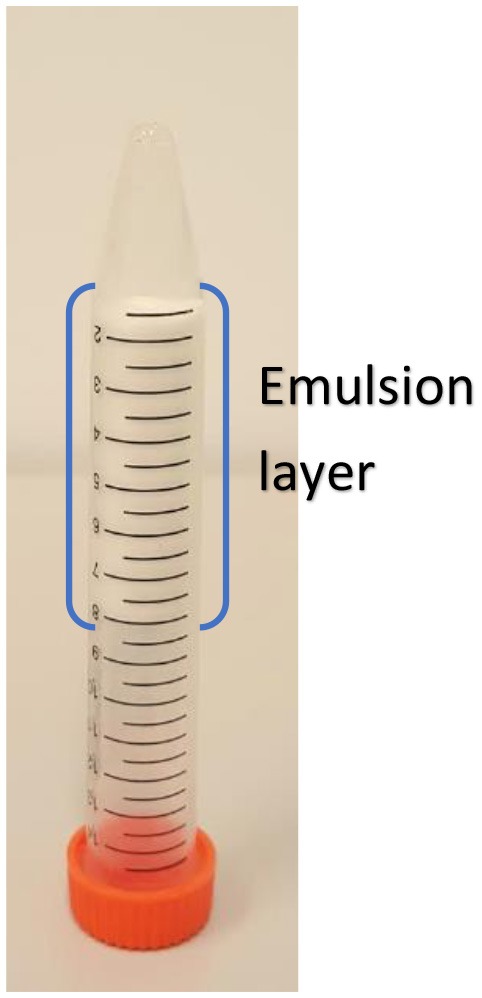
Photograph of C. Oil/water emulsion with 3 wt% CNC and 100 mM NaCl after centrifugation.

Salt greatly affects the stability of C. Oil/water emulsions. Salts are well known for screening the electrostatic repulsion on CNC surfaces (Kalashnikova et al., 2012). The zeta potential of CNC decreased linearly from (–)70 mV to (–)50 mV with the addition of 0 to 3 mM NaCl (Figure 7). The limiting conditions of CNC based Pickering emulsions in the presence of NaCl were studied (Figure 8). Experiments were initially conducted with the addition of 100 Mm NaCl solution to varying CNC concentrations ranging from 3 to 0.1 wt%. After identifying that 0.1 wt% CNC can stabilize C. Oil/water emulsions with 100 mM NaCl, the NaCl concentration was varied, keeping the CNC concentration constant at 0.1 wt%. It was found that CNC could stabilize C. Oil/water emulsions at 0.1 wt% concentration with as little as 3 mM NaCl concentration. Emulsion volume remained constant for NaCl concentration ranging from 3 to 20 mM. As the NaCl concentration increased from 20 to 50 mM, the emulsion volume increased by 15%. At 1 wt% CNC concentration, the emulsion volume is 2.5 mL which is 3 times higher than in absence of salt. Interestingly, the emulsion volume at 3 wt% CNC and 100 mM NaCl concentration correspond to the total volume of the aqueous suspension and oil used. Even with the centrifuge tube placed upside down, the emulsion after stability test did not flow, forming a gel-like network (Figure 9). Rheological properties of this gel are discussed in the last part of the discussion section.

**Figure 10:**
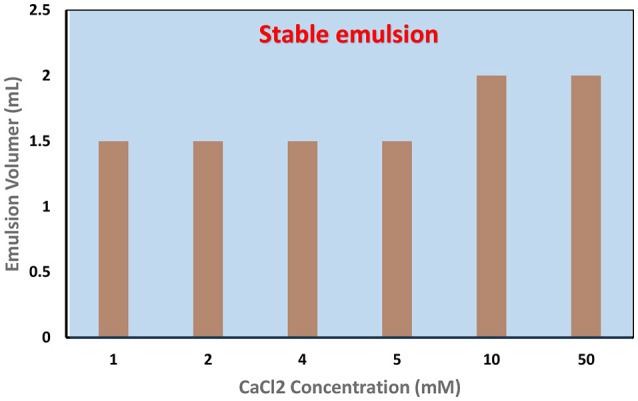
Effect of CaCl_2_ addition on the stability of CNC (0.1 wt%) stabilized C.Oil/Water emulsions.

Similarly, the effect of CaCl_2_ addition on the stability of C. Oil/water emulsion was tested (Figure 10). It was found that CNC could stabilize C. Oil/water emulsions at 0.1 wt% concentration with CaCl_2_ concentration as low as 1 mM or even lower.

### Effect of pH on C. oil/water emulsions stability

**Figure 11:**
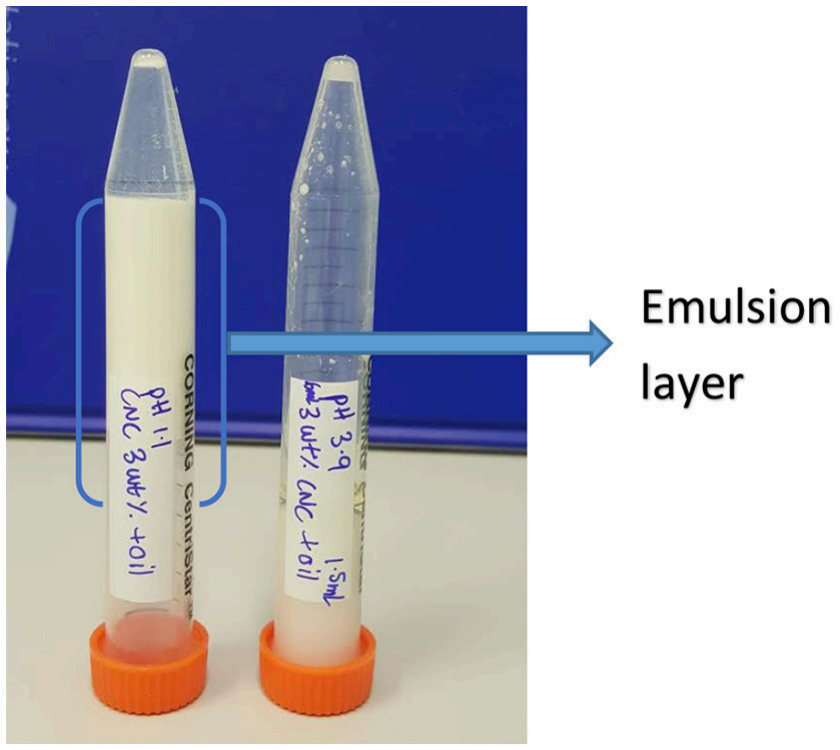
Photograph of C. Oil/water emulsion with 3 wt% CNC and pH 1.1 and 3.9.

Varying pH from 7 to 3 had no effect on the stability of C. Oil/water emulsions (Table 1). 0.1 wt% CNC could not form stable C. Oil/water emulsions for pH ranging between 7 and 3. In contrast, at pH 2 or below, 0.1 wt% CNC formed stable emulsions. On the other hand, 3 wt% CNC could form stable C. Oil/water emulsion at pH 7 or below. At pH below 2, the emulsion volume represented the combined volume of the aqueous suspension and oil used. The emulsion prepared with 3 wt% CNC at pH 3.9 flowed when the centrifuge tube was turned upside down; the emulsion formed with 3 wt% CNC at pH 1 did not. At lower pH, the emulsion formed a gel network (Figure 11).

**Table 1:** Effect of pH on the stability of C. Oil/water emulsions.

**CNC concentration (wt%)**	**pH**	**Emulsion condition**	**Emulsion Volume (mL)**
0.1	7	No Emulsion	0
	6	No Emulsion	0
	5	No Emulsion	0
	4	No Emulsion	0
	2	Stable	2
	1.1	Stable	2
3	3.9	Stable	2
3	1.1	Stable	9

**Figure 12:**
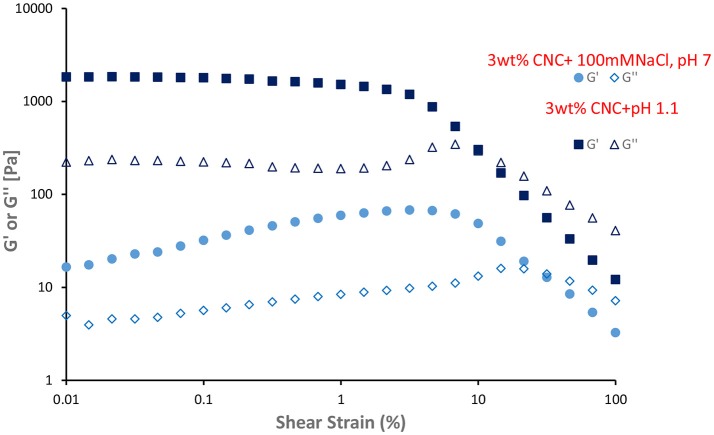
Dynamic strain sweep of gel like emulsions (3 wt% CNC and 100 mM NaCl, 3 wt% CNC and pH 1.1) at 25°C and frequency of 1 Hz. Filled symbol indicate elastic moduli (G') and unfilled symbol indicate loss moduli (G”).

**Figure 13:**
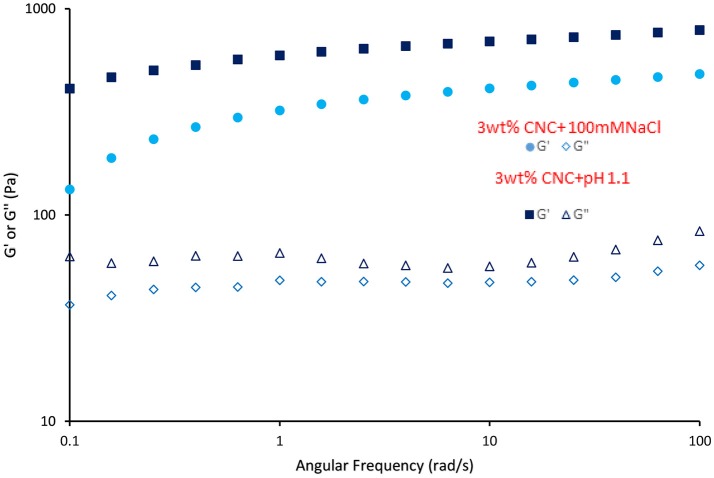
Dynamic frequency sweep of gel like CNC emulsion (3 wt% CNC and 100 mM NaCl, 3 wt% CNC and pH 1.1) at 25°C and 0.1% strain. Filled symbol indicate elastic moduli (G') and unfilled symbol indicate loss moduli (G”).

The rheological properties of the C. Oil in water gel are shown in Figures 12, 13. Gel-like emulsions are subjected to strain (Figure 12) and frequency (Figure 13) sweeps. In a strain sweep, the range of viscoelastic behavior can be quantified for gels. The elastic modulus G' describes the solid-like behavior of gel whereas the loss or viscous modulus G” defines the liquid-like behavior of the material.

## Discussion

Particle concentration is an important factor in the formation of particle stabilized emulsions, commonly known as Pickering emulsions. It has a remarkable influence on the emulsion stability. Stable Pickering emulsion formation is a two-step process. Firstly, particles migrate from the aqueous dispersion onto the oil–water interface. Then, particles adsorb at the oil/water interface replacing and decreasing the oil-water contact area. The adsorbed particles act as emulsifiers, by lowering the interfacial free energy primarily by reducing the interfacial area between the two phases and makes the system stable (Wang, 2013).

The amphiphilic nature of negatively charged CNC's is the driving force for their migration from solution toward the oil/water interface during emulsification. However, significant electrostatic repulsion occurs when a negatively charged CNC (because of sulfate ester groups on the surface) approaches the oil-water interface that is also negatively charged due to the preferential adsorption of hydroxide ions (Capron and Cathala, 2013). Such repulsion can create an energy barrier preventing particle adsorption to the interface, and hinder the formation of emulsions (Danov et al., 2005; Golemanov et al., 2006). Hence, stable emulsions could not form at low concentrations of CNCs ranging from 0.1 to 0.5 wt% (Figures 2, 3).

At higher concentrations, starting from 1 wt%, CNC tend to aggregate in aqueous solution (Xu et al., 2017) because of chemical interaction (for example hydrogen bond) and strong affinity toward material containing hydroxyl groups (hydrogen bonding between CNC and water molecules) (Li et al., 2015). CNC aggregates have higher adsorption energy (force of attraction) that dominates the repulsion force between CNCs and oil/water interface (Paunov et al., 2002). Adsorbed CNC aggregates minimize the interfacial free energy primarily by reducing the interfacial area between the oil/water interfaces. Hence, CNC's can stabilize the emulsions at higher concentrations than 1 wt%.

### Effect of salts addition on emulsion stability

The amount of CNC required to stabilize the emulsion reduced greatly with the addition of either NaCl or CaCl_2_. Figure 7 shows that the zeta potential of CNC suspension reduced gradually with increasing NaCl concentration. Zhong et al. (2012) and Prathapan et al. (2016) reported similar observations. This is because of the electrostatic screening effect from the cation counter ion Na^+^ and the Debye-Huckel screening strength augments upon increasing salt concentration; therefore, the Debye length decreases. For example, the Debye length and ionic strength of NaCl at 1 mM concentration are 9.6 nm and 1 mol/m^3^, respectively; at 3 mM concentration, these are 5.54 nm and 3 mol/m^3^. Prathapan et al. (2016) further reported that Ca^2+^ ions screen stronger than Na^+^ ions. Hence, there was a greater reduction in CNC zeta potential with the addition of Ca^2+^ ions. The Debye length of CaCl_2_ at 1 mM concentration is 4.52 nm, which is lower than that of NaCl at 3 mM concentration (5.54 nm).

Double layer principles imply that the adsorption of ions and ion pairs to the CNC surface shield the surface charge of CNC and reduces the electrostatic repulsion, which facilitates the CNC to migrate and adsorb onto the oil/water interface in the presence of Na+ and Ca2+ ions, even at 0.1 wt% CNC concentration. Hence, a stable C. Oil/water emulsion could form at 0.1 wt% CNC in the presence of Na+ and Ca2+ ions. The amount of CNC required to stabilize 2 mL of C. Oil is 30 times less in the presence of salts. Interestingly, to stabilize C. Oil/water emulsion with a 0.1 wt% CNC suspension, < 1 mM CaCl_2_ addition to the CNC suspension is required, which is 3 times lower than for NaCl. This directly corroborates the Debye length of CaCl_2_ and NaCl. These results indicate that stabilization of oil/water emulsion with CNC is governed by the surface charge in the presence of electrolytes.

Zhong et al. reported that a 0.15 wt% CNC suspension is clear and transparent, indicating a stable suspension because of charge repulsion among CNC's (Zhong et al., 2012). When ionic strength increased to 50 mM Na^+^, CNC particles tend to aggregate to a size of 980 nm, which is 10 times higher than their initial size. This is because of van der Waals forces dominating the electrostatic repulsion. Xu et al. reported that CNC tends to aggregate even in the absence of electrolytes at concentrations higher than 1 wt% (Xu et al., 2017). When the suspension concentration is higher than 3 wt%, aggregation of CNC became denser through long-range electrostatic interactions (Xu et al., 2017). When 100 mM NaCl is added to a 3 wt% CNC suspension, CNC aggregates grew in size and connected into a percolating network. Macroscopically, gel-like behavior is observed. Peddireddy et al. also reported similar observations with CNC concentrations higher than 12 g/L and 70 mM NaCl (Peddireddy et al., 2016). Emulsions prepared with a 3 wt% CNC suspension and ionic strength of 100 mM Na^+^ behave like gels. The emulsion volume is 9 mL, corresponding to the total volume of oil and aqueous suspension.

### Effect of pH on emulsion stability

Prathapan et al. (2016) and Zhong et al. (2012) reported that when the pH is varied from 11 to 2, the change in CNC suspension zeta potential is very low. This is because pH did not significantly alter the disassociation state of the sulfate ester groups present on the CNC surface since the pK_a_ of covalently bound sulfate ester group (pK_a_ = 1.9) is very low. However, for pH below 2, a considerable reduction in zeta potential–i.e., net charge on CNC surface, and agglomeration of CNC results because of the protonation of the sulfonic acids starting to occur. Again, reduction in the net charge of CNC at pH below 2 helps their migration and adsorption at the oil/water interface to form stable emulsions with 0.1 wt% CNC. When emulsions are prepared with 3 wt% CNC at pH 1.1, aggregates of CNC grew in size and connect into a percolating network gel.

Emulsions prepared with 3 wt% CNC, 100 mM NaCl, and pH 1.1 show gel behavior. Viscoelastic properties of these gels were presented in Figures 12, 13. G' and G” of gel-like emulsions obtained at pH 1.1 are higher than those with 100 mM NaCl. At low shear stresses, both gel emulsions possess a linear viscoelastic region (LVR) wherein the elastic modulus G' and viscous modulus G” are independent of shear stress. Within this region, G' is dominant over G”, indicating that the material is acting as a solid; elastic behavior dominates over viscous comportment. At a critical shear stress, the gel yields as shown by the decrease in G', and then reaches a “cross-over point” where G” becomes dominant and the gel begins to flow. Past this critical stress, the viscous regime dominates (G” > G') indicating that the network structure has yielded and begins to behave as a non-Newtonian shear thinning fluid. Figure 13 gives the frequency sweep i.e., the time-dependent behavior of the gel emulsion. For both gel emulsions, the G' and G” values are non-intersecting with G' increasing gradually with angular frequency. This slight increase in moduli shows these gels to be weakly cross-linked (Mendoza et al., 2018).

## Conclusion

Cellulose nanocrystals (CNC) can form stable oil/water emulsions regardless of their charge density. This contradicts the limiting condition of charge density below 0.033 e/nm^2^ reported in the literature for the formation of stable emulsions with CNC. However, higher charge density CNC requires higher amounts of CNC and higher concentrations. At high CNC concentrations, the adsorption forces of CNC aggregates dominate the electrostatic repulsion between CNC and the oil/water interface. As the charge density reduces through the addition of salts, lower concentrations of CNC are required because of the salt induced reduction in electrostatic repulsion. The amount of CNC required to stabilize 2 mL of oil is 30 times less in the presence of salts. The ratio of minimum ionic strength required to prepare a stable emulsion with CaCl_2_ and NaCl directly corroborates with the Debye length ratio of CaCl_2_ and NaCl. This indicates that stabilization of oil/water emulsion with CNC is governed by surface charge in the presence of electrolytes. Emulsions prepared with 3 wt% CNC suspensions have an ionic strength of 100 mM Na^+^ and behaved like a gel. Varying pH from 7 to 3 had no effect on the stability of emulsions. However, the emulsion prepared with 3 wt% CNC and at pH below 2 behaved like a gel. These gels are weakly cross-linked. Cellulose nanocrystal offers a new way to stabilize oil in water by forming Pickering emulsions. The systems are completely biodegradable and biocompatible and can form robust gels or not, opening new innovation avenues in food and biomedical applications.

## Author contributions

SV, WB, and GG prepared manuscript. SV, LH, and LM performed experiments. RP and RT performed AFM measurements.

### Conflict of interest statement

The authors declare that the research was conducted in the absence of any commercial or financial relationships that could be construed as a potential conflict of interest.
